# *Calligonum polygonoides* L. as Novel Source of Bioactive Compounds in Hot Arid Regions: Evaluation of Phytochemical Composition and Antioxidant Activity

**DOI:** 10.3390/plants10061156

**Published:** 2021-06-06

**Authors:** Mukesh K. Berwal, Shravan M. Haldhar, Chet Ram, Sandip Shil, Ramesh Kumar, Jagan S. Gora, Dhurendra Singh, Dilip K. Samadia, Manoj Kumar, Mohamed Mekhemar

**Affiliations:** 1 Division of Crop Improvement, ICAR–Central Institute for Arid Horticulture, Bikaner 33400, India; haldhar80@gmail.com (S.M.H.); chetram.nbpgr@gmail.com (C.R.); rameshflori@gmail.com (R.K.); jagangora@gmail.com (J.S.G.); dhuren6@gmail.com (D.S.); samadiadk@yahoo.com (D.K.S.); 2 Department of Entomology, Central Agriculture University, Imphal 795004, India; 3 Department of Social Sciences, ICAR–Central Plantation Crops Research Institute, Research Centre Mohit Nagar, Jalpaiguri 735102, India; sandip.iasri@gmail.com; 4 Chemical and Biochemical Processing Division, ICAR—Central Institute for Research on Cotton Technology, Mumbai 400019, India; 5 Clinic for Conservative Dentistry and Periodontology, School of Dental Medicine, Christian-Albrecht’s University, 24105 Kiel, Germany

**Keywords:** antioxidant activity, total phenolic content, seasonal variation, *Calligonum polygonoids*

## Abstract

*Calligonum polygonoides* L. (Phog) is an endemic perennial herb that is highly resistant to all type of abiotic stresses and dominant biomass as well as phytochemicals producer in its natural habitat of the “Thar Desert” of Rajasthan, India. The present study was conducted to evaluate the effect of extreme environmental conditions on the phenolic, flavonoids, tannin content, and total antioxidant activities of *C. polygonoides* foliage harvested during different months. It exhibited a significant variation in the content of phenolic compounds, flavonoids, tannins, and antioxidant activity with harvesting time and all parameters are positively correlated to each other. The highest phenolic compounds and antioxidant activity was observed during severe winter and summer months, when monthly average environmental temperature was lowest and highest of the year, respectively. On the basis of the results, two harvests of *C. polygonoides* foliage during June and December are advised to maximize the phenolic compound production with highest antioxidant activity. These results demonstrate *C. polygonoides,* which is a dominant biomass producer under the harsh climatic conditions, can be an important source for the development of the functional foods rich in antioxidants in hot arid regions.

## 1. Introduction

Phenolic compounds are plant secondary metabolites, which are constituents of both edible and non-edible parts of plants [1]. They are considered active substances in plant and play a role in plant growth or defense against competitors, pathogens, or predators as well as abiotic stress and have beneficial effects on human health due to their biological activity like anticancer, antioxidant, anti-inflammatory, and antimicrobial activities [2,3]. Phenolic compounds possess the antioxidant activity through free radicals scavenging by donating hydrogen atoms or electron or by chelating metal ions [4,5]. In the ever-changing scenario, awareness toward healthy and balance eating habit to cope with stressful everyday life has attracted people towards natural antioxidant-rich foods instead of synthetic antioxidants [6,7].

*Calligonum polygonoides* L. is an abiotic stress-tolerant perennial shrub of sand-dunes eco-system and well-known for its energy-rich fuel-wood, leaf-fodder, and flower-buds. It grows well under resource-poor conditions where any type of vegetation is not possible. *C. polygonoides* is a dominant biomass producer under extremes of concurrent abiotic stresses at sandy areas of the Thar Desert [8]. Under rainfed cultivation, 6–7 years old plant gives about 14.85 kg biomass annually including flower bud, foliage, and fuel wood. The foliage of *C. polygonoids,* which is an extremely rich source of phenolic compounds, is also a byproduct, since almost 70–80% foliage dropped down during the month of December–January and behaves deciduous. During last week of February, when night temperature crosses 12 °C, new flesh starts coming along with flowers [9,10]. Plants showed quick growth, huge flowering, and seed formation from March–May month, and this is the period for bio-mass harvest through phogala collection, looping of foliage, and light pruning. The plant starts re-sprouting during the on-set of monsoons and foliage may be ready for looping in November–December. These flower buds convert to seed which matures in the month of May–June. If the plant is pruned (seeds and Foliage) during the month of June, post monsoon new flush starts showing up in the months of August–September. In this way, almost double biomass can be harvested from the *C. polygonoids* plant [9]. *C. polygonoides* has high economic values as all its plant parts are utilized in different purposes. All its plant parts are highly rich sources of phenolic compounds and possessed 13–35% phenolic compounds on dry weight basis with major portion of gallic acid, catechin vanillic, chlorogenic acid, epicatechin, coumaric acid, catechol, vanillic acid, epicatechin, and syringic acid [11]. Samejo et al. [12] reported the presence of different secondary metabolites viz., phenolics, flavonoids, tannin, steroids and terpenoids in different parts of phog plant and its higher scavenging activity against 2,2-diphenyl-1-picrylhydrazyl (DPPH), 2,2′-azino-bis(3-ethylbenzothiazoline-6-sulfonic acid (ABTS), and superoxides and also identified some flavonoid compounds in flower buds [13,14,15]. Recently, Berwal et al. [16] reported very high antioxidant activity along with the presence of many phytochemicals like furan-2,5-dimethyl, 2,3-dihydro-3,5-dihydroxy-6-methyl-4H-Pyran-4-one (DDMP), dehydromevalonic lactone, deoxyspergualin, 2-methoxy-4-vinylphenol, benzeneethanol-4-hydroxy-, quinic acid, lauric acid, linolenic acid, and squalene in flower buds (phogala) with scientifically proven bio-activities like anti-microbial, anti-inflammatory, anticancer, anti-diabetic, hepatoprotective, cardiovascular, antioxidant, and anti-mutagenic.

The phytochemicals obtained from plants are a source of raw material for the production of nutraceuticals and pharmaceutical products, which has an enormous therapeutic application to relieve certain sufferings that affect human health [17,18,19,20,21,22,23]. Economical and safe production of these plant-based bioactive compounds are the main challenges since under natural conditions production of these bioactive compounds in plant system (except some medicinal and aromatic plants) is relatively less (less than 1% of dry weight) and depends greatly on the physiological and developmental stage along with the environmental factors such as temperature, light intensity, soil water, soil salinity, and fertility. Therefore, the field of interest is the optimization of production level of these bioactive compounds in plants.

**Figure 1:**
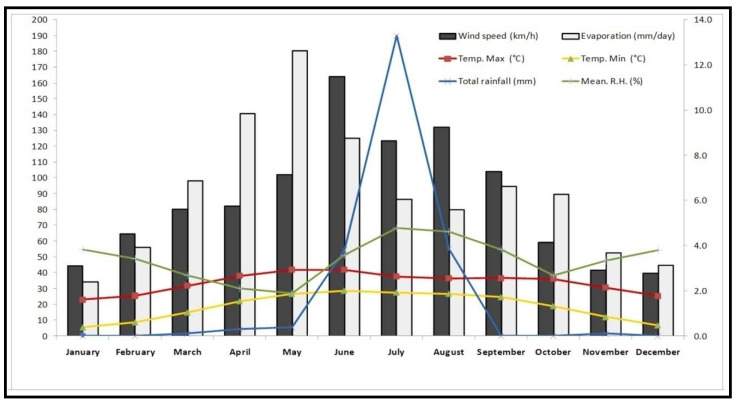
Month wise average weather data of Bikaner district during the study period (January to December, 2018).

The hot arid region of the “Thar Desert” has all extremes in climatic conditions with very low annual rain fall (<350 mm), very hot summers with monthly maximum average temperature to >40 °C and some days it touches 50 °C with very high UV radiations (May–June), and extremely cold winters with monthly average minimum temperature to as low as 5 °C and some days it goes subzero °C in December–January (Figure 1). To even survive under these extreme climatic conditions, plant has to have an in-built specific ROS scavenging mechanism to fight with the incoming huge oxidative stresses. *C. polygonoids* not only survive under these extreme climatic conditions but also produce a significant amount of biomass.

In the present study, effect of variations in climatic conditions on antioxidant activity, phenolics, flavonoids, and condensed tannin content was investigated in *C. polygonoides* foliage harvested in Thar Desert of India. Various plants produce secondary metabolites (phenolic compounds) to safeguard themselves from abiotic stressors and at the same time phenolic bioactives can be extracted and utilized for the formulation of functional foods with high antioxidant activity. *C. polygonoides* herb can grow well in both high and low temperature stress under arid conditions. Hence, it is crucial to know the variation in phenolic compounds and the antioxidant activity of the extracts from *C. polygonoides* foliage in accordance with the seasonal variation. As far as available literature, this investigation is the first report on *C. polygonoides* considering the effect of climatic conditions or seasonal variation on phytochemical profile and antioxidant activity. Consequently, the determination of secondary metabolite profile with seasonal variation, could provide crucial knowledge on the time of harvest of foliage that afford optimum concentration of active ingredients for functional food formulations.

## 2. Results and Discussion

### 2.1. Seasonal Variations of Total Phenolic Content (TPC), Flavonoids (TFC), and Tannin Content (TTC)

TPC, TFC, and TTC were estimated following the standard protocols in methanolic extracts of *C. polygonoides* foliage harvested during different months from plants grown under hot arid region. All three parameters viz., TPC, TFC, and TTC varied significantly (*p* < 0.05) along the years with harvesting months. The significant difference in these secondary metabolites with harvesting month is due to the differences in environmental conditions which has a great influence on biosynthesis and accumulation of these compounds in the plants [24,25]. The highest values for TPC, TFC, and TTC were recorded during the peak summer (June) and peak winter (December) period.

**Figure 2:**
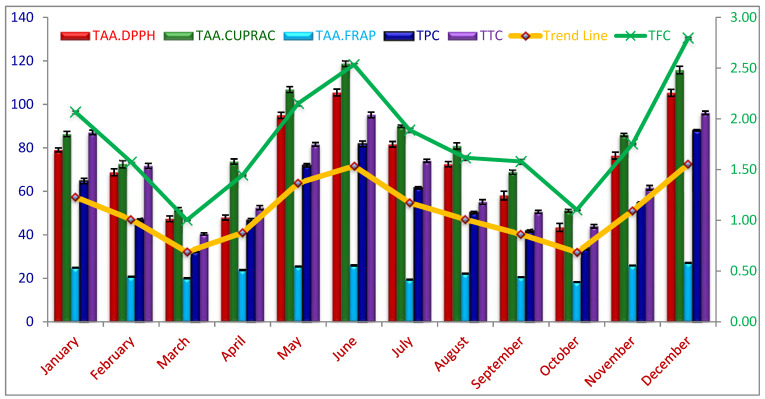
Changes in total phenolics, flavonoids, tannin, and total antioxidant activity of *Calligonum polygonoides* L. Foliage during different seasons under extreme climatic condition. Data are presented as mean ± SD, n = 3 experiments, *p* < 0.05.

The seasonal fluctuations in TPC of *C. polygonoides* foliage was expressed as GAE using the standard curve equation; obtained results are given in Table 1 and Figure 2. A significant difference was observed among TPCs recorded during different months (*p* < 0.05). TPC of *C. plygonoides* foliage, harvested during different months ranged from 32.28 ± 0.54 to 88.08 ± 0.59 mg.GAE.g^−1^ FW. TPC content in foliage is lower than that of previous reports of Berwal et al. [11], with 151 mg.GAE.g^−1^ TPC in *C. polygonoides* foliage because the authors reported values on dry weight basis while our results are on fresh weight basis. Samejo et al. [12] also reported higher values for TPC in *C. polygonoides* plant. The highest TPC was recorded during December month, reaching to 88.08 ± 0.59 mg.GAE.g^−1^ FW followed by June, January, and May with 81.84 ± 2.28, 71.97 ± 1.33, and 64.75 ± 2.13 mg.GAE.g^−1^ FW respectively. The lowest TPC was observed during the month of March and October with a magnitude of 32.28 ± 0.54 and 34.05 ± 1.53 mg.GAE.g^−1^ FW, respectively which are statistically at par to each other (*p* < 0.05).

The seasonal variations in TFC can be clearly seen from the Table 1 and Figure 2. A significant variation was observed in the TFC values (*p* < 0.05), varying from 1.0 ± 0.01 to 2.80 ± 0.02 mg.CtE.g^−1^ FW. Like TPC, the highest TFC was also recorded during the month of December (2.80 ± 0.02 mg.CtE.g^−1^ FW), followed by June (2.53 ± 0.03), May (2.15 ± 0.04), and January (2.07 ± 0.03); and lowest TFC was recorded during March (1.0 ± 0.01) and October (1.1 ± 0.01). The highest TFC values are lower than that of the previous reports. Berwal et al. [11] reported about 6.5 mg.CtE.g^−1^ TFC in *C. plygonoides* foliage on dry weigh basis.

The tannin content (TTC) of *C. polygonoides* foliage was expressed as mg.Catechin.E. g^−1^ FW, depicted in Table 1 and Figure 2. The TTC of *C. polygonoides* foliage harvested during different months significantly varied from each other (*p* < 0.05) with a magnitude of 40.40 ± 0.89 to 96.09 ± 1.38 mg.Catechin.E.g^−1^ FW. Similar to TPC and TFC, the highest TTC was observed in December (96.09 ± 1.38) followed by June (95.17 ± 2.22) and January (87.13 ± 1.78) while lowest during the month of March (40.4 ± 0.89) and October (43.94 ± 1.40) mg. Catechin.E.g^−1^ FW.

### 2.2. Seasonal Variations of Total Antioxidant Activity

The total antioxidant activity (TAA) of methanolic extract of *C. polygomoides* foliage harvested during different months was determined based on its reducing capacity by different methods such as CUPRAC, FRAP, and DPPH assay and expressed as mg ascorbic acid equivalent per g (mg.AAE.g^−1^) FW. Generally, these three assays attributed consistent results with the seasonal changes of TPC and TFCs (Figure 2). Determinations of reducing power in amalgamation of different methods helps in comprehending the real nature of the antioxidant compounds present in the foliage of *C. polygomoides* [26]. TAA of *C. polygomoides* foliage harvested during different months varied significantly (*p* < 0.05) under all assay procedures followed (Table 1 and Figure 2). It was observed that TAA of methanolic extract of *C. polygomoides* foliage varied significantly (*p* < 0.05) with harvesting months irrespective of the assay procedure followed. The highest TAA was observed during June and December followed by May and January, while the lowest during March and October months under all assay procedures.

The TAA of *C. polygomoides* foliage harvested during different months determined under CUPRAC assay varied significantly (*p* < 0.05) from 51.22 ± 1.14 to 118.84 ± 2.12 mg.AAE.g^−1^ FW (Table 1 and Figure 2). The highest TAA was observed during June (118.84 ± 2.12 mg.AAE.g^−1^) followed by December (115.81 ± 2.97 mg.AAE.g^−1^), while the lowest in October and march with a magnitude of 51.22 ± 1.14 and 51.73 ± 1.42 mg.AAE.g^−1^, respectively and significantly at par with each other.

The TAA of *C. polygomoides* foliage harvested during different months recorded under FRAP assay also fluctuated significantly (*p* < 0.05) from 18.26 ± 0.21 to 27.11 ± 0.20 mg.AAE.g^−1^ FW. The highest TAA recorded under FRAP assay was during the month of December (27.11 ± 0.20 mg.AAE.g^−1^) followed by June (25.97 ± 0.41 mg.AAE.g^−1^) while the lowest during October and July with a magnitude of 18.26 ± 0.21 and 19.42 ± 0.23 mg.AAE.g^−1^ respectively.

Similarly, the TAA of *C. polygomoides* foliage estimated under DPPH scavenging activity varied along the year with harvesting months. The results showed that TAA was significantly (*p* < 0.05) different among harvesting months with a magnitude of 43.42 ± 3.14 to 105.46 ± 2.70 mg.AAE.g^−1^ FW (Table 1 and Figure 2). Similar to FRAP assay, the highest TAA was observed during the month of December (105.46 ± 2.72 mg.AAE.g^−1^) which was significantly at par with June (105.30 ± 2.72 mg.AAE.g^−1^), while the lowest during the month of October and March with the magnitude of 43.42 ± 3.14 and 47.39 ± 2.21 mg.AAE.g^−1^ FW.

### 2.3. Principal Component Analysis (PCA)

**Figure 3:**
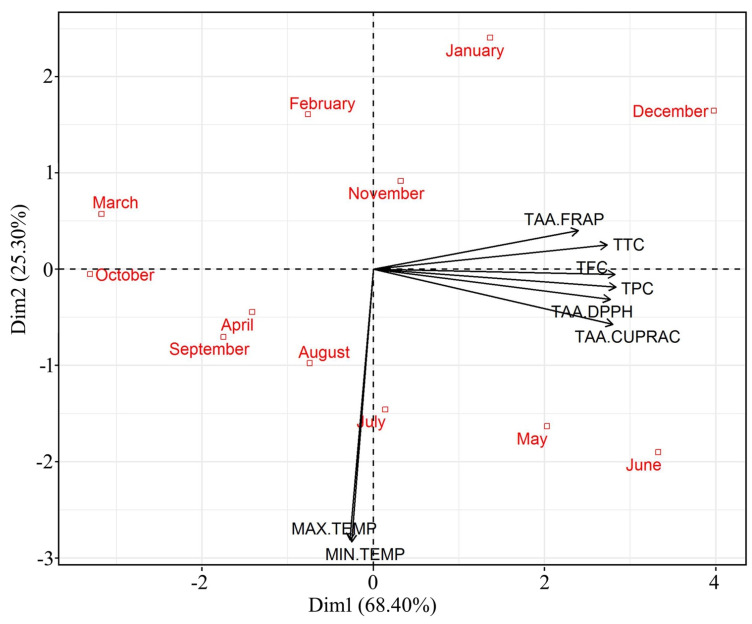
Principal component analysis (PCA) of TAA, phenolics, flavonoids, and tanning content in *C. polygonoides* foliage during different months.

The PCA carried out with the assayed parameters like TAAs, phenolic, flavonoids, and tannin content, explained 93.7% of variability in axis one and two. The PC1 explained 68.4% of the combined variance and the second component (PC2) explained 25.3% (Figure 3). This clearly demonstrated that there is a variability in the parameters assayed in relation to the sampling months or environmental temperature. The level of all parameters (TAA, phenolics, flavonoids, and tannin contents) was strongly related to axle one. The biplot graph from this analysis confirmed the occurrence of seasonality in antioxidant responses in *C. polygonoides* foliage, marked by all the assayed parameters like TAA, phenolic, flavonoids, and tannin contents (Figure 3). The sampling units of summer months (May and June) and winter months (Deceber and January) were grouped on the positive side of axle one and characterized by the highest values for TAA, phenolics, flavonoids, and tannin content. On the other hand, the sampling units of the remaining months were generally grouped at the opposite side of this axle. Plants generally produce more antioxidants under oxidative stress conditions and in the hot arid region, the environmental conditions are highly toward extreme sides. During summer months the environmental temperature remains as high as 48 °C with very high radiations and winter with extremely low temperature reaching to subzero °C. Similar results were also reported in *Ipomoea nil* cv. Scarlet O’Hara [27].

PC1 Equation: depicting power of antioxidants

(1) PC1=0.423∗TPC+0.408∗TTC+0.413∗TAA.DPPH+0.422∗TFC+0.418∗TAA.CUPRAC+0.358∗TAA.FRAP−0.041∗MAX.TEMP−0.038∗MIN.TEMP

### 2.4. Loess Regression Analysis against Maximum and Minimum Temperature

**Figure 4:**
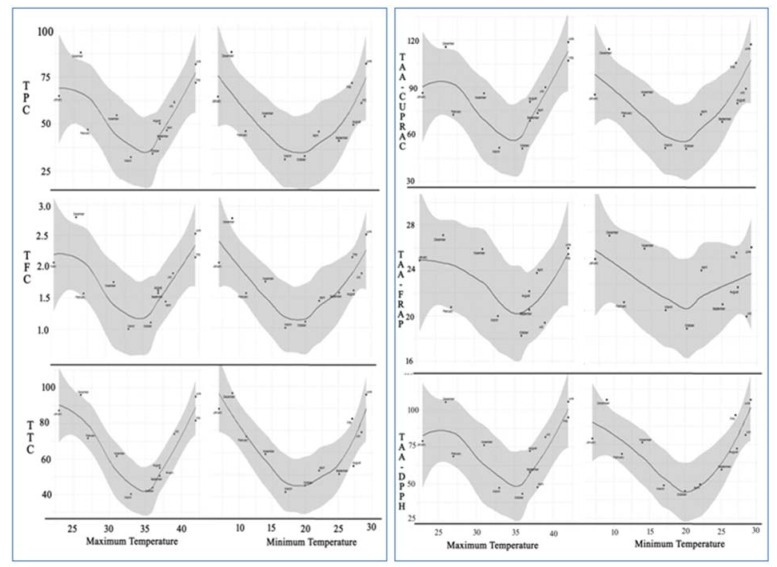
LOESS regression fitting of seasonal variation in TPC, TFC, TTC and total antioxidant activities (TAA.CUPRAC) of *C. polygonoides* foliage against maximum and minimum average monthly temperature.

To visualize the seasonal changes in TPC, TFC, TTC, and antioxidant activity, locally weighted scatter-plot smoother (LOESS) curve was plotted for data recorded during the twelve sampling times against the maximum and minimum monthly average temperature (Figure 4). Resultantly, we received a “V” shaped regression curve from all parameters studied along with the fluctuations in maximum and minimum temperature from 35 ± 1.0 °C to 20 ± 1.0 °C, respectively.

From the regression curve, it was observed that *C. polygonoides* plant quickly responded to the changes in maximum and minimum temperatures toward both sides through changes in biosynthesis and accumulations in TPC, TFC, TTC, and antioxidant compounds in arial parts. With respect to the months, December–January and May–June are plotted at top of both the arms of “V” for all the parameters. This is because of the extreme environmental temperature that occurred, like very hot summers with monthly maximum average temperature going beyond 42 °C and some days 50 °C in May–June months and extremely cold winters with monthly average minimum temperature going as low as 5 °C and some days beyond zero °C in December–January months. As discussed in the introduction part that the phenolic compounds and antioxidants play a vital role in plant defense against oxidative stresses that occurred in plants as a result of biotic or abiotic environmental stimuli [2,3]. Similarly, *C. polygonoides* produced a plethora of phenolic compounds and antioxidants to cope with the oxidative stress due to the stimuli produced in plants by extreme temperatures during the discussed seasons.

### 2.5. Correlation between Contents of Phenolic Compounds and Antioxidant Activity

In order to determine the possible relationship between TPC, TFC, TTC, and total antioxidant activity of *C. polygonoides* foliage, Pearson’s correlation analysis was conducted based on the results observed for these parameters at twelve sampling times (Table 2). A strong correlation between TPC, TFC, TTC, and total antioxidant activity in CUPRAC, FRAP, and DPPH assays was observed. Our result is in accordance with the recent reports, which suggested that higher phenolic content exhibited stronger antioxidant activity. A similar linear correlation was also reported in *Moringa oleifera, Cyclocarya paliurus* leaves, *Juglans sigillata* husk, *Ocimum basilicum* leaves, and *Juglans regia* [17,28,29,30,31]. Moreover, the correlation coefficient between phenolic compounds and total antioxidant activity in CUPRAC and DPPH assay (r^2^ > 0.91, *p* < 0.05) is stronger than that of antioxidant activity in FRAP assay (r^2^ < 0.80, *p* < 0.05). These results indicate that phenolics and flavonoids are major bioactive compounds in *C. polygonoides* foliage, which impart maximum antioxidant activity.

## 3. Discussion

Phenolics, flavonoids, and tannins are secondary metabolites and are distributed ubiquitously in the plant kingdom [32,33] and possess numerous biological activities like antioxidant activity, anti-inflammatory, antibacterial activities etc., [2,34,35,36]. Recent literature has demonstrated the uniqueness of the plant extracts and their compounds with exceptional biomedical and food applications [37,38,39,40,41,42]. These compounds also play a very vital role in plant defense mechanism against different types of biotic as well as abiotic stresses [43,44]. The biosynthesis and accumulation of secondary metabolites like phenolics and flavonoids in plants are greatly influenced by intrinsic and extrinsic factors. The intrinsic factors include genetic make-up and physiological condition of the plant while extrinsic factors include biotic (insect, pest, and diseases) and abiotic (high and low temperature, availability of light and water, soil properties etc.,) environmental stimuli which occurs during the growing period of the plant [24,25,44,45,46]. In our study, the level of phenolic compounds and antioxidants in *C. polygonoides* foliage was continuously increased with increasing as well as decreasing in average maximum temperature from 35 ± 1.0 °C and average minimum temperature of 20 ± 1.0 °C. In other words, the lowest level of phenolics and antioxidants was observed during the month of March and October when the monthly average maximum and minimum temperature stands near 35 ± 1.0 °C and 20 ± 1.0 °C, respectively while the highest values were recorded during the months of December–January and May–June, when the monthly average max. and min. temperatures were lowest <25.3 and <7 °C and highest >42.0 and >28.8 °C respectively of the whole year. The temporal variation on phenolic compounds and antioxidants in *C. polygonoides* foliage presented here are partly in tune with the previous results. Cao et al. [28] attributed the highest values for phenolic and flavonoids compounds in *C. paliurus* during the month of May–July and November and, Amaral et al. [25] attributed the increase in phenolic compounds and antioxidants in walnut during the month of July when the solar radiation level was at highest. Similarly, Deng et al. [47] in *C. paliurus* and Tsormpatsisidis et al. [48] in Lollo Rosso lettuce, reported the increased accumulation of total flavonoids under higher radiations.

It is a well-known fact that plants also produce active oxygen species (AOSs) during their regular metabolic processes but the level of its production increased multifold under environmental stresses. There are increasing evidences that phenolics and flavonoids act as antioxidants under certain physiological conditions and, thereby, responsible for protecting the plants from damage caused by these AOSs generated under oxidative stresses caused by very high and low temperature, radiations etc. Hence, exposure to very high and very low temperature stimulated the synthesis and accumulation of these shielding compounds like phenols, flavonoids, and other antioxidants, especially in epidermis of fully developed leaves [43,44,49,50].

Many previous studies have confirmed that primary and secondary metabolisms in plant share common precursors and intermediate, which create competitions for common precursors between phenolic biosynthetic pathways and growth [51,52,53]. Cao et al. [28] reported reduced accumulations of phenolics in *C. paliurus* leaves during June, which is the reproductive and active growth phase of *C. paliurus.*

Ma et al. [54] also reported a significant decline in quercetin contents in *Apocynum venetum* and *Poacynum hendersonii* leaves after flowering because a considerable amount of photosynthates diverted toward reproductive organs when plants enter the reproductive stage. Similarly, in *C. polygonoides*, reduced accumulation of phenolics and flavonoids during March and October implies that higher amount of photosynthates are allocated for reproductive growth during March and active growth during October months, Samadia et al. [9]. With the onset of these two growth phases, the accumulation of these compounds starts increasing in *C. polygonoides* foliage and reached to maximum during June and December month respectively. As discussed in introduction part, sprouting of new flesh in *C. polygonoides* occurs during February just after shedding of foliage in December–January and, if looping has been carried out in June month than re-sprouting of new flesh also occurs during the month of September [9]. Taking into consideration the results of this study and facts discussed above, it can be concluded that to maximize the phenolic production from *C. polygonoides* foliage, two times foliage harvesting should be carried out during the months of June and December, when the phenolic accumulation occurs to maximum.

## 4. Material and Methods

### 4.1. Experimental Site and Plant Material

**Figure 5:**
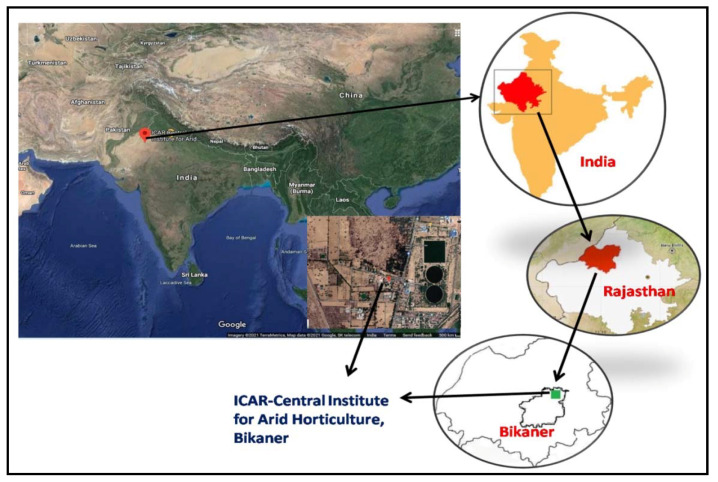
Location map of the experimental site (250 07′ 080 N 730 20′ 44′ E and 217 m above mean sea level) satellite and schematic view.

This experiment was carried out selecting similar looking 5–6-years-old plant of *C. polygonoides* grown at the research farm of ICAR—Central Institute for Arid Horticulture, Bikaner, Rajasthan which falls under the western dry zone of India located at 250 07′ 080 N and 730 20′ 44′ E, 217 m above mean sea level (Figure 5). This region has classic arid climate with extreme hot and dry summer (40–50 °C) followed by cold winter (0–3 °C). The mean annual rainfall of the experimental site was 300–373 mm during 2017 to 2019 (Figure 4). The soil type was virgin aridisol with a pH of 8.1 and EC (1:2) of 0.80 dS m^−1^, a cation exchange capacity (CEC) 12.73 cmol kg^−1^, organic carbon 0.08 g kg^−1^, soil N 75.23 kg ha^−1^, P 12.65 kg ha^−1^, and K 325.90 kg ha^−1^. *C. polygonoides* foliage used in this study was collected from plants grown completely under rainfed condition. The samples were collected in liquid nitrogen in the second week of every month from January to December, 2018 from the above-mentioned experimental site and transported to the plant biochemistry department and stored at −80 °C till further use.

### 4.2. Chemicals

All the chemicals used in this study were purchased from Sigma-Aldrich (St. Louis, Missouri, United States), Merck India (Mumbai, India) and Hi-Media (Mumbai, India).

### 4.3. Sample Extraction

Accurately weighed 500 mg *C. polygonoids* foliage samples store at −80 °C was ground to fine powder with liquid nitrogen and homogenized in 10 mL of 70% methanol with the help of a mortar and pestle and incubated at 70 °C in water bath for 1 h. Use of liquid nitrogen reduces the changes that happened in bioactivities due to heat generated by corrosion effect in mortar and pestle during grinding [55]. After incubation the tubes were subjected to centrifugation at 10,000 rpm for 10 minutes at 4 °C, the supernatant was collected. The residue was re-extracted twice with 5 mL of 70% methanol and centrifuged. The supernatant was pooled and made up the volume to 20 mL with extraction solvent and stored at −20 °C till further use.

### 4.4. Total Phenolic Content (TPC)

TPC in different solvent extracts was estimated following Folin-Ciocalteu method described by Berwal et al. [11]. TPC in different solvent extracts was expressed as mg gallic acids equivalents (GAE). g^−1^ fresh weight (FW). All the extracts were analyzed in triplicates.

### 4.5. Total Flavonoids Content (TFC)

TFC in different solvent extracts was estimated by aluminum chloride-based colorimetric method described by Medini et al. [56]. A volume of extracts (1 mL) was mixed with 0.3 mL each of 5% NaNO_2_ and 10% AlCl_3_ and 3.4 mL of 1 M NaOH. The resultant reaction mixtures were incubated for 15 minutes at room temperature and the OD was measured at 510 nm against the reagent blank. The total flavonoids content was expressed as catechol equivalent (Ct.E). g^−1^. The whole assays were carried out in triplicate in order to get mean value.

### 4.6. Tannin Content (TTC)

Condensed tannin content was determined by following the methods described by Rebaya et al. [57], using catechin as a reference standard. A volume of 1 mL methanolic extract was added to 3 mL of 4% vanillin in methanol and 1.5 mL concentrated hydrochloric acid. After incubation for 15 min at room temperature under dark, the absorbance was recorded at 500 nm and the condensed tannin content was expressed as mg.Catechin.E. g^−1^ FW.

### 4.7. Total Antioxidant Activity (TAA)

TAA of methanolic extract of *C. polygonoides* was determined on four different methods namely cupric reducing antioxidant capacity (CUPRAC), ferric reducing antioxidant power (FRAP and DPPH) assay with following the standard methods. The reducing capacity of the extracts was assayed by CUPRAC method described by Apak et al. [58] with minor adjustments. In this assay, simultaneously 1 mL each of cupric chloride (10 mM), ethanolic neocuproine (75 mM), and ammonium acetate (1 M, pH 7.0) were mixed in test tubes containing 1.9 mL of distilled water and 100 µL ethanolic extracts. These mixtures were incubated in dark for 30 min at room temperature and the OD was measured at 450 nm against the reagent blank. Ascorbic acid was used as the positive reference standard and the results were expressed as mg.AAE.g^−1^ FW. Whole assays were carried out in triplicate in order to get mean value.

TAA by FRAP assay was carried out following the method described by Benzie and Strain [59] with some modifications. Ethanolic extract (100 µL) of *C. polygonoides* foliage was added separately in test tubes containing 2.9 mL of FRAP working reagent. The fresh FRAP working reagent was prepared by mixing of 300 mM acetate buffer (pH 3.6), 10 mM TPTZ (2,4,6-tripyridyl-s-triazine) in 40 mM HCl and 20 mM FeCl_3_.6H_2_O solution in 10:1:1 ratio. The reaction mixture was allowed to react under dark for 30 min. The absorbance of the colored complex (ferrous tripyridyl triazine complex) was taken at 593 nm. Ascorbic acid was taken as positive reference standard. All assays were carried out in triplicate and averaged.

The DPPH scavenging assay was done according to the method of Berwal et al. [11]. Ethanolic extracts of *C. polygonoides* foliage (100 μL) was allowed to react with 2.9 mL of 0.006% ethanolic DPPH for 10 min under dark condition. A control was also run simultaneously with 100 μL distilled water instead of extract. The absorbance was taken at 517 nm. Ascorbic acid was used as reference standard (10–50 μg/mL). Whole assay was carried in five replicates and averaged.

### 4.8. Statistical Analysis

The generated assays were carried out in triplicate and various statistical parameters (like mean-variance statistic, PCA, biplots, regression, etc.,) were computed using R software version 3.6.0 [60]. To study the regression effect of temperature on various parameter estimates, locally weighted scatter-plot smoother (LOESS) regression technique was adapted [61,62].

## 5. Conclusions

In conclusion, the results of the present study evidently demonstrated that *C. polygonoides* foliage possessed very higher phenolics as well as antioxidant activity. Both phenolic compounds and antioxidant activity exhibited a significant seasonal variation. The strong positive correlation between phenolic compounds and antioxidant activities indicates that phenolic compounds are major antioxidant compounds in *C. polygonoides* foliage. The harvest season variation in phenolic content and antioxidant activities is solely dependent on the environmental temperature and the highest values were observed during severe winter and summer months. Based on the results, two harvest of *C. polygonoides* foliage is advised, first during the month of June and second during the month of December to achieve maximum yield of bioactive compounds of phenolic yield. The present study gives the clue about the new insights of concurrent abiotic stresses in molecular farming for production of bioactive compounds for nutraceuticals and pharmaceutical industry through arid horticultural flora specifically *C. polygonoids* under hot arid region of Rajasthan. This strategy can be a boon for local rural farming community for improving their socio-economic status with livelihood security.

## Figures and Tables

**Table 1:** Seasonal variation in TPC, TFC, TTC and total antioxidant activity in CUPRAC, FRAP, and DPPH assay of *C. polygonoides* foliage. Different letters indicate the significant difference between harvesting times for the same category according to Duncan’s test (*p* < 0.05). Values are mean of three replications.

Sampling Month	TPC (mg.GAE.g^−1^)	TFC (mg.CtE.g^−1^)	TTC (mg.catechin.E.g^−1^)	Total Antioxidant Activity (mg.AAE.g^−1^)		
				CUPRAC	FRAP	DPPH
January	64.75 ^d^	2.07 ^d^	87.13 ^b^	86.38 ^cd^	24.88 ^c^	79.06 ^c^
February	46.91 ^f^	1.57 ^g^	71.73 ^d^	72.59 ^e^	20.77 ^f^	68.75 ^de^
March	32.28 ^h^	1.00 ^j^	52.48 ^f^	51.72 ^f^	20.02 ^fg^	47.39 ^g^
April	46.70 ^f^	1.45 ^h^	40.40 ^g^	73.70 ^e^	23.77 ^d^	47.95 ^g^
May	71.97 ^c^	2.15 ^c^	81.57 ^c^	106.82 ^b^	25.46b ^c^	94.93 ^b^
June	81.84 ^b^	2.53 ^b^	95.17 ^a^	118.84 ^a^	25.97 ^b^	105.46 ^a^
July	61.61 ^d^	1.89 ^e^	74.05 ^d^	90.00 ^c^	19.42 ^g^	81.65 ^c^
August	50.26 ^f^	1.62 ^g^	55.12 ^f^	80.89 ^d^	22.18 ^e^	72.46 ^e^
September	41.87 ^g^	1.58 ^g^	50.56 ^f^	68.88 ^e^	20.56 ^f^	58.09 ^f^
October	34.05 ^h^	1.10 ^i^	43.94 ^g^	51.22 ^f^	18.26 ^h^	43.42 ^h^
November	54.66 ^e^	1.75 ^f^	61.64 ^e^	86.02 ^cd^	25.88 ^b^	76.42 ^de^
December	88.08 ^a^	2.80 ^a^	96.09 ^a^	115.81 ^a^	27.11 ^a^	105.30 ^a^

TPC: total phenolic content; TFC: flavonoids content, TTC: tannin content; CUPRAC: CUPric reducing antioxidant capacity; FRAP: ferric reducing antioxidant power. DPPH: 1,1-diphenyl-2-picrylhydrazyl; GAE: gallic acid equivalent, CtE: catechol equivalent, AAE: Ascorbic acid equivalent.

**Table 2:** Pearson correlation coefficients among TPC, TFC, TTC, and total antioxidant activity (CUPRAC, FRAP, and DPPH) of *C. polygonoides* L. foliage at twelve sampling times.

	TPC	TFC	TTC	TAA.CUPRAC	TAA.FRAP	TAA.DPPH
TPC	1	0.99 **	0.95 **	0.98 **	0.79 **	0.96 **
TFC		1	0.94 **	0.97 **	0.78 **	0.95 **
TTC			1	0.91 **	0.70 *	0.93 **
TAA.CUPRAC				1	0.80 **	0.97 **
TAA.FRAP					1	0.73 *
TAA.DPPH						1

* and ** indicate the significance at the 0.05 and 0.01 level, respectively. TPC—total phenolic content; TFC—total flavonoids content, TTC—total tannin content; CUPRAC–CUPric reducing antioxidant capacity; FRAP: ferric reducing antioxidant power. DPPH: 1,1-diphenyl-2-picrylhydrazyl.

## Data Availability

Data available on request.
